# Development of Insulin Resistance through Induction of miRNA-135 in C2C12 Cells

**DOI:** 10.22074/cellj.2016.4563

**Published:** 2016-08-24

**Authors:** Maryam Honardoost, Ehsan Arefian, Masoud Soleimani, Sara Soudi, Mohammad Reza Sarookhani

**Affiliations:** 1 Department of Molecular Medicine, School of Medicine, Qazvin University of Medical Sciences, Qazvin, Iran; 2 Stem Cell Technology Research Center, Tehran, Iran; 3 Department of Microbiology, School of Biology, College of Science, University of Tehran, Tehran, Iran; 4 Department of Hematology, Faculty of Medical Sciences, Tarbiat Modares University, Tehran, Iran; 5 Department of Immunology, Faculty of Medical Sciences, Tarbiat Modares University, Tehran, Iran

**Keywords:** Insulin Resistance, MiR-135, Insulin Receptor, C2C12

## Abstract

**Objective:**

Micro-RNAs (miRNAs) are a class of posttranscriptional regulators that play crucial roles in various biological processes. Emerging evidence suggests a direct link between miRNAs and development of several diseases including type 2 diabetes (T2D). In this study, we aimed to investigate the effect of predicted miRNA and target genes on insulin resistance.

**Materials and Methods:**

This experimental study was conducted on the C2C12 cell line. Using bioinformatics tools miRNA-135 and two respective target genes-insulin receptor (*Insr*) and vesicle associated membrane protein 2 (*Vamp2*)were selected as potential factors involved in insulin resistance process. Levels of glucose uptake miRNA expression and respective gene targets were determined after cell transfaction by miR-135.

**Results:**

It was determined that *Insr* gene expression was significantly down-regulated in miR-135 transfected C2C12 cell line (P≤0.05). Interestingly; these transfected cells have shown a significant difference in glucose uptake incomparision the positive control cells, while it was similar to the insulin resistant cell line (P≤0.05). In contrast, no significant alteration of *Vamp2* gene expression was observed.

**Conclusion:**

Our data indicated no change on the *Vamp2* expression level after miRNA transfection, while expression level of *Insr* was reduced and miR-135 expression was contrarily increased leading to poor stimulation of glucose uptake through insulin, and development of insulin resistance phenotype in C2C12 cell line.

## Introduction

Micro-RNAs (miRNAs) are small noncoding RNA molecules composed of 21-23 nucleotides regulating gene expression through inhibition or degradation of 3´UTR of target mRNA (1). Several evidences indicate on the pivotal roles of these biological molecules in a wide variety of physiological processes and diseases (2,5). Thus, it is not surprising that the discovery of entirely new class of gene expression regulators prompted several research teams to investigate the potential effect of miRNAs on the development of diabetes and its complications (6,7). Now a day, growing evidences indicate that miRNAs are involved in the pathogenesis of type 2 diabetes (T2D) (7,8). 

T2D is a less defined condition, culminating in dysregulation of blood glucose levels due to development of insulin resistance and/or relative insulin deficiency. Insulin resistance, as a major component of T2D disease, is thought to result mainly from the various environmental factors including obesity (8). 

Skeletal muscle insulin resistance is the earliest visible metabolic defect during the onset of T2D disease (9,10). Additionally, skeletal muscle insulin resistance could lead to the other metabolic syndrome related deregulations (11,12). Therefore, further understanding of skeletal muscle insulin resistance mechanisms is imperative. 

Diverse mechanisms have been identified as underlying factors responsible for skeletal muscle insulin resistance, such as genetic components (13,14). Using different approaches to identify the genes associated with T2D, more than 30 genes have determined contributing to insulin resistance, most of which could affect insulin signaling pathway (15). 

Alternatively, detection of miRNAs, as important metabolic regulators, have highlighted a novel regulatory mechanism of action suggesting a possible role of these RNA molecules in diabetes. Consistent with previous studies demonstrating contribution of very specific miRNAs in various metabolic processes associated to T2D, it is proposed that miRNAs play critical roles during the inception and progression of this complex metabolic disease (16,18). 

Although unusual miRNA signatures have been reported with regards to diabetes, the status and role of these miRNAs have yet remained to be elucidated in the diabetic skeletal muscle. Therefore, an exhaustive investigation of miRNA roles in the diabetic skeletal muscle is necessary (19). It has been demonstrated that insulin resistance was developed in skeletal muscle cell lines, including C2C12 muscle cells (20,21). Therefore, this cell line could be used to screen the potential compounds like miRNAsinvolved in insulin resistance of skeletal muscle. 

**Fig.1:**
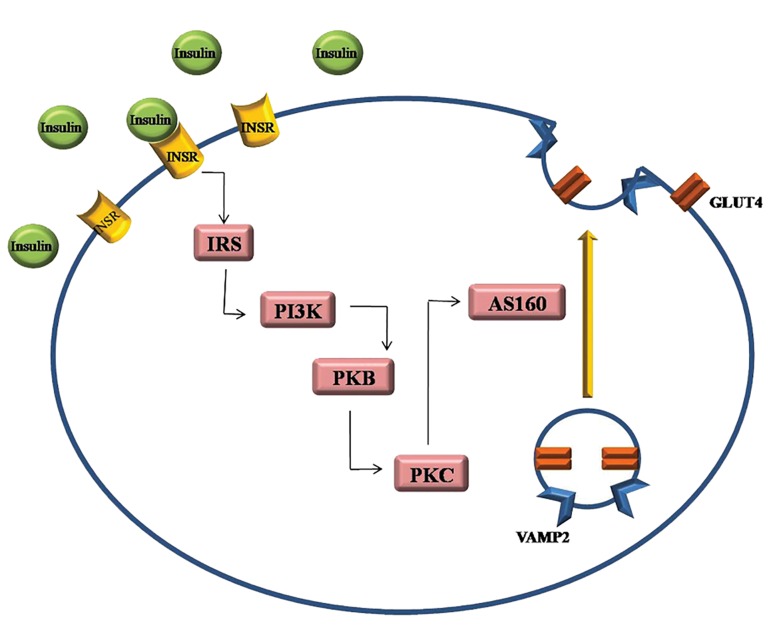
Insulin receptor signal transduction via PI3K/PKB pathway. Insulin activates tyrosine kinase, which phosphorylates and recruits different substrate adaptors such as the IRS protein family. Tyrosine-phosphorylated IRS subsequently displays binding sites for numerous signaling partners, among which PI3K has a major role in insulin function, mainly via the activation of the PKB and the PKC pathways. It stimulates glucose uptake in the muscles via translocation of GLUT4 vesicles to the plasma membrane through phosphorylation of AS160. Under basal conditions, small positive GLUT4 vesicles also contain VAMP2. VAMP2 is involved in the docking and fusion of GLUT4 vesicles with the plasma membrane and indeed, studies have implicated a role for VAMP2 in insulin-dependent trafficking in cell. PI3K; Phosphatidylinositol-4,5-bisphosphate 3-kinase, PKB; Protein kinase B, IRS; Insulin receptor substrate, PKC; Protein kinase C, GLUT4; Glucose transporter type 4, VAMP2; Vesicle-associated membrane protein 2, AS160; AKT substrate of 160 kDa, and INSR; Insulin receptor.

In this study, we bioinformatically focused on miR-135 and two predicted target genes, insulin receptor (*Insr*) and vesicle associated membrane protein 2 (*Vamp2*) as the first components in the cascade causing insulin resistance in cells. Insulin receptor signal transduction has been shown in Figure 1. Decrease in *INSR* expression has been described in the obese insulin resistant patients with T2D, as remarkably hyperinsulinemic individuals (15,22). 

Insulin helps glucose transportation by promoting the exocytosis of the glucose transporter type 4 (GLUT4) through plasma membrane. Delivery of GLUT4 to the plasma membrane is mediated by formation of functional complexes containing *VAMP2* that mediates cAMP-stimulated exocytosis in endocrine cells. Delivery of GLUT4 has been indicated to be impaired in the disease states of insulin resistance and T2D (23). 

In this study, we aimed to investigate the effect of predicted miRNA (miR-135) and two respective target genes in development of insulin resistance. We have provided evidences that miR-135 directly induces insulin resistance in C2C12 cell line by targeting the insulin signaling pathway. 

## Materials and Methods

### Cell culture

With regards to presence of large amount of the total insulin mediating glucose uptake, this experimental study was conducted on the C2C12 cell line obtained from skeletal muscle, as one of the major insulin target tissues (24). 

C2C12 myoblasts line (obtained from Stem Cells Technology Research Center, Tehran, Iran) were cultured in growth medium (GM) composed of Dulbecco’s Modified Eagle Medium (DMEM, Gibco, UK), 10% fetal bovine serum (FBS, Gibco, UK), penicillin 100 IU/ml and streptomycin 100 μg/ml (Gibco, UK) followed by incubation at 37˚C and 5% CO_2_ , before starting differentiation process. Upon reaching cell density to 70%, they were digested with 0.25% trypsin and seeded into culture dishes. As soon as obtaining more than 90% confluent dish, C2C12 myoblast cells differentiation procedure was initiated by changing GM to differentiation medium (DM) containing DMEM supplemented with 3% horse serum (Gibco, UK). According to previous studies, 70% confluent C2C12 cells were converted in differentiation medium in absence of insulin sensitive cell (IN) or in chronic presence of 100 nM insulin (Gibco, UK) resistant cell (IRC) for 3 days. 

### Immunocytochemistry

After inducing myogenic differentiation, the cultured C2C12 cells in 12-well plates were washed with PBS and fixed with 4% paraformaldehyde for 15 minutes. 0.5% Triton X-100 was used for permeabilization. The cells were then blocked in 2% goat serum (Sigma, USA) diluted in phosphatebuffered saline (PBS). After blocking, the cells were incubated with anti-PAX7 or anti-myosin primary antibody (Sigma, USA) at 37˚C for 1-2 hour(s). Subsequently, the cells were washed and, the secondary fluorescent antibodies (Ray Biothech, USA) were added to the cells at 37˚C for 1 hour. The nuclei were ultimately stained with DAPI (Invitrogen, USA) for 30 seconds. 

### Glucose uptake study

In this study, untreated differentiated C2C12 cells (NCC) were incubated in DMEM culture media and considered as negative control. In contrast, differentiated C2C12 cells treating with 1 mM insulin (Sigma, USA) DMEM culture media, during the glucose uptake procedure, was used as positive control. Differentiated C2C12 cells treated with 100 nM insulin for 72 hours or transfected with miR-135 during differentiation process were utilized as experimental cells. After treatment, a glucose uptake assay was performed. Cells were incubated for 1 hour in glucose and serum-free media followed by 3 hour incubation in DMEM containing 5 mM glucose in the presence and absence of 1 mM insulin. Based on Gallant et al. (25) study, after insulin exposure, 50 ml of the media aliquots were taken from the respective wells and added to 150 ml distilled water to achieve four times dilution. The remaining glucose in the media was quantified using the COBAS INTEGRA Glucose HK GEN.3 kit (Roche, Germany). A glucose standard curve was constructed using glucose concentrations ranging between 0.25 mmol and 2 mmol (4.5 mg/dl and 36 mg/dl), which was measured spectrophotometrically at 340 nm (data not shown). All standard curve concentrations were determined by triplicate values. 

### Target prediction and pathway analysis

According to a wide range literature review and KEGG database (www.genome.jp/kegg), we categorized a set of significantly important genes in insulin resistance pathway. Using Target Scan 6.2, miRWalk and RNAhybrid, we generated a list of miRNA candidates containing a seed site for the previously categorized genes (26,28). Those genes were predicted as miRNA targets by at least two of three prediction databases. Based on bioinformatics prediction, we found that miR-135 targeted several biological molecules, which are regulator of insulin signaling pathway such as *Insr* and *Vamp2*, as an important component of glucose transporter type 4 (Gut4) translocation pathway (Fig.1). 

### Construction of miR-135 expression vector

To create pre-miR expression vectors, we first amplified a 400 bp DNA fragment covering a premiR, using mouse genomic DNA as a template. PCR reactions were performed corresponding specific primers and then amplified fragment was cloned into a lentiviral vector (pCDH-CMVMCSEF1-copGFP from System Biosciences, USA) at XbaI and EcoR1 sites. Partial digestions were performed in cases that there was an internal XbaI and EcoR1 site to obtain the DNA fragment carrying the pre-miR. Expression of mature miR was confirmed by the TaqMan real-time PCR kit (Applied Biosystems, USA) (29) and bidirectional sequencing (Microgene, Korea). 

### Transfection of miR-135

C2C12 cells were transfected immediately after trypsinization, while the cells were in suspension with the scramble or miR-135 mimic (pCDH/miR135) using Lipofectamine 2000 (Invitrogen, USA). The 70% confluent C2C12 cells were trypsinized and then seeded in 6-well plates in growth medium. Immediately after plating the cells, while they were still in suspension, differentiation medium was supplemented with the complexes and added to the cells. The cells were incubated with lipid-DNA complexes, while they were attached to the culture dish at 37˚C and 5% CO^2^.The complex medium was removed after 24 hours followed by adding fresh differentiation medium. Ultimately, the cells and respective media were collected at 72-hour post-transfection of the cell differentiation and subjected to reverse transcriptase-polymerase chain reaction (RT-PCR) and glucose uptake analyses. 

### RNA isolation and quantitative reverse transcriptase-polymerase chain reaction

Total RNA was extracted from the cells using TRIzol (Invitrogen, USA) according to the manufacturer’s instruction. The quality and concentration of total RNA were subsequently estimated using denatured gel electrophoresis and spectrophotometer, respectively. According to previous study miR could be amplified with stem-loop realtime PCR using specific stem-loop primers (30). Expression level of the corresponding predicted mRNA targets (*Insr* and *Vamp2*) to the selected miR was also validated by real-time PCR using gene specific primers. The threshold cycle average was used for data analysis by Rotor-gene Q software (Corbett, Australia). Genes and related specific primers are presented in the Table 1. *Insr* and *Vamp2* expressions were normalized against the expression of *β-actin. Snord-6 (U6)* was also selected as internal control for miR expression. All PCRs were performed in triplicates and run in at least 3 independent experiments. The 2^−ΔΔCt^algorithm was employed to evaluate the relative expression level of each gene. 

**Table 1:** Designed specific primers for PCR assays


Primer	Sequence (5´-3´)

Reverse transcription	
miR 135astem-loop	5ˊGTCGTATGCAGAGCAGGGTCCGAGGTATTCGCACTGCATACGACTCACA 3ˊ
qRT-PCR	
miR-135a	F : 5ˊCGATATGGCTTTTTATTCCTA3ˊ
	R : 5ˊGAGCAGGGTCCGAGGT 3ˊ
*Insr*	F: 5ˊAACAGATGCCACTAATCCTTC 3ˊ
	R: 5ˊGCCCTTTGAGACAATAATCC 3ˊ
*Vamp2*	F: 5ˊGTCACTGCCTCTGCCAAG3ˊ
	R: 5ˊGTCCACCACCTCATCCAC3ˊ

qRT-PCR; Quantitive reverse transcriptase-polymerase chain reaction.

### Comparison of quantitative reverse transcriptase-polymerase chain reaction result by a high throughput method data 

We next investigated whether the candidate gene expression levels obtained by quantitative RTPCR (qRT-PCR) were comparable with other methods. To this end, the qRT-PCR results of candidate genes were compared with two microarray data Gene Expression Omnibus (GEO) including muscle samples from insulin resistance individuals (GEO accession # GSE6798) and blood samples from T2D patients (GEO accession # GSE26168) (31,32). 

### Statistical analysis

The data are presented as mean GS.E.M. To determine statistical significance, student’s t test was applied. The significance threshold was set at P≤0.05. All experiments were performed tree times. 

## Results

### Characterization of C2C12 differentiation

**Fig.2:**
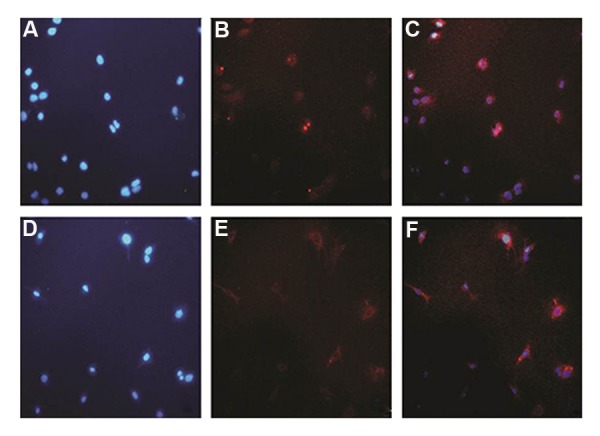
Myoblast to myocyte differentiation using 3% horse serum. A-C. C2C12 myoblasts were stained with PAX and DAPI antibodies, as positive control of precursor cells. After that myoblasts were differentiated with DMEM medium containing 3% horse serum during 3 days and D-F. The differentiated cells were seeded in new plate and stained with (MyHC) antibody as well as DAPI. DAPI; 4´,6-diamidino2-phenylindole and DMEM; Dulbecco’s Modified Eagle Medium.

C2C12 cells were proliferated in the presence of serum and differentiated upon partial serum deprivation from myoblast cells causing their differentiation into myocytes. This was validated by positive Immunocytochemistry (ICC) result for specific skeletal marker, myosin. Using ICC technique, we also determined C2C12 myoblast cell type expressing precursor cells marker, PAX7 (Fig.2). 

### In vitro studies on miR135-transfected C2C12 cells 

#### Quantitative reverse transcriptase-polymerase chain reaction analysis

**Fig.3:**
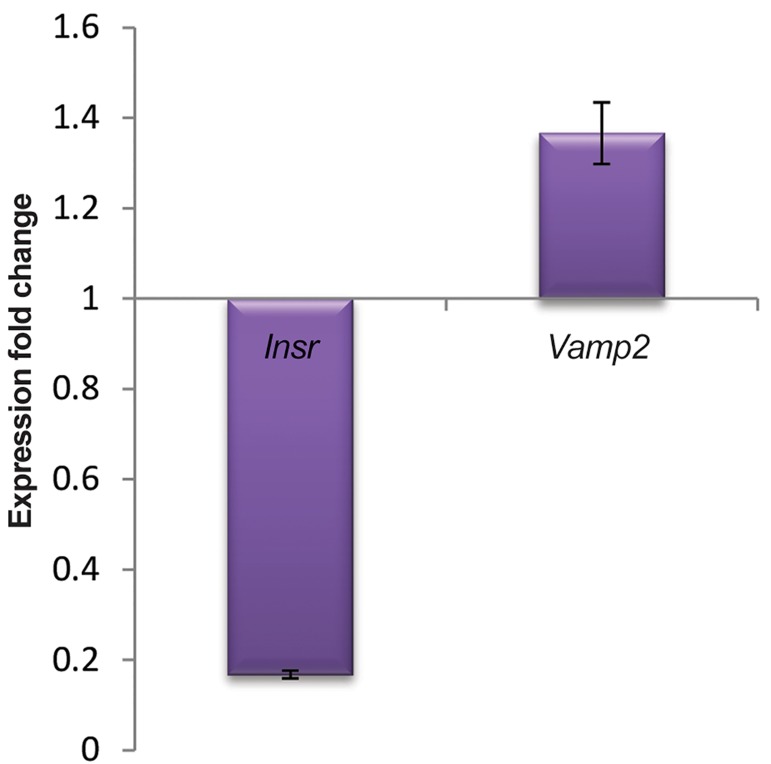
Expression pattern of the candidate genes during miR-135 transfection. Based on real time (qRT-PCR) results which were concurrent to miR-135 up-regulation in transfected cells, Insr has significant down-regulated profile during differentiation process (P≤0.05). In contrast, no significant overexpression of Vamp2 was observed (P>0.05). Error bars indicate SEM (n=3). qRT-PCR; Quantitive reverse transcriptase-polymerase chain reaction.

qRT-PCR analysis showed that miR-135 was induced and had significantly altered expression pattern in miR-transfected C2C12 cells compared to negative control cell line scramble (miR-135 fold change: 1.537, P≤0.05). We have examined *Vamp2* and *Insr* expressions in transfected C2C12 cells, to identify potential markers within development of insulin resistance (Fig.3). Interestingly, qRT-PCR analysis, showed that expression level of *Insr* targeted by miR-135 in silico, was down-regulated in transfected cells (*Insr* fold change: 0.168, P≤0.05). There was a significant decrease in *Insr* gene expression in the transfected C2C12 cells compared to negative control, indicating a down-regulation of *Insr* gene expression due to the miR-135 transfection. In contrast to *Insr* expression level in transfected C2C12 cells, miR-135 expression level was upregulate (P≤0.05), suggesting this miR is responsible for the decreased level of *Insr*. No significant alteration was observed on the expression of *Vamp2* (*Vamp2* fold change: 1.366, P>0.05). 

### Sustained expression of miR-135 attenuated glucose uptake in C2C12

Insulin was shown to provide a significant increase in the amount of glucose taken up by positive control cell (PCC). 

Our PCC, showed a significant increase in glucose uptake in C2C12 cells (G uptake: 48 mg/dl) compared to the negative control (NCC, G uptake: 32 mg/dl, P≤0.05). 

**Fig.4:**
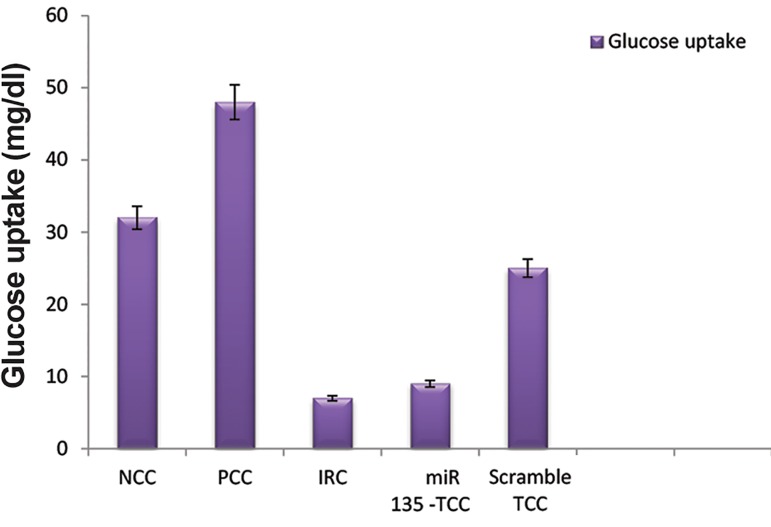
Image illustrates glucose uptake in different status of C2C12 cells. IRC and TCC showed a reduction in G uptake compared to PCC. Error bars indicate SEM (n=3). NCC; Normal control negative, PCC; Normal control positive, IRC; Insulin resistance cell, and TCC; miR-135-trasfected Cell.

Hyperinsulinemia was used to induce insulin resistance in the C2C12 cells (IRC, G uptake: 7 mg/ dl). Findings showed a significant suppression of glucose uptake in the IRC compared to the PCC (Fig.4). 

Transfected C2C12 (TCC) where exposed for 72 hours to differentiation medium. Thereafter glucose uptake was determined. Interestingly, the miR-135-trasfected cells (G uptake: 9 mg/dl) showed a significant difference in glucose uptake in comparison with PCC (P≤0.05), while it was similar to IRC (P=0.71). 

### Evaluation of reverse transcriptase-polymerase chain reaction result by microarray data

To extend the results of qRT-PCR, we used Affymetrix cDNA microarray row data (GEO accessions # GSE6798 and GSE26168) and analyzed relative transcript levels *Vamp2* and *INSR* in control samples compared to insulin resistance in muscle and blood samples (31,32). 

**Fig.5:**
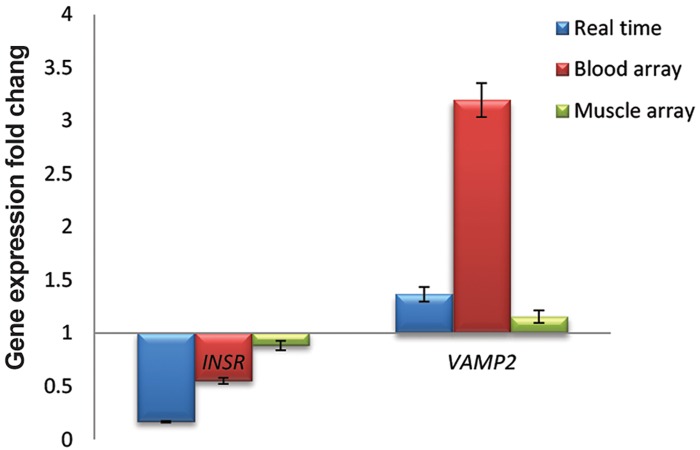
Comparison of miR-135 predicted gene target expression levels during myogenesis using two different methods. Fold changes in qRT-PCR and two microarrays were compatible for INSR as well as qRT-PCR Muscle microarray for VAMP2 (P≤0.05). Error bars indicate SEM (n=3). qRT-PCR; Quantitive reverse transcriptase-polymerase chain reaction.

Data analysis reflected similar down-regulation trends for *INSR* expression obtained from both microarray results; muscle samples (*INSR* fold change: 0.884, P≤0.05) and blood samples (*INSR* fold change: 0.552, P≤0.001). This was similar to our observation obtained from qRT-PCR analysis (Fig.5). On the contrary, there was an up-regulation for *VAMP2* expression in all three studies. Muscle microarray analysis showed a significant up-regulation for *VAMP2* (*VAMP2* fold change: 1.156, P=0.036), although no significant change was observed for *VAMP2* expression in blood sample (*VAMP2* fold change: 3.1949, P=0.271) in comparison with healthy control (Fig.5).

## Discussion

The present study investigated association of the miR-135 and relative targets to insulin resistance *in vitro*, with the aim to understand the processes involved in pathogenesis of T2D. It has been reported that miRs can potentially be served as novel regulator of diseases, including T2D (6,14). This theory was supported by Kosaka et al. (33) which generated the idea of miRs-mediated intercellular statement. Although there are several reports describing the status and role of miRs in diabetic tissues, very few studies have focused on the diabetic skeletal muscle (7,8,17,18,34). 

The principal objective of present study was based on the fact that defects in the insulin signaling pathway leads to T2D and insulin resistance is crucial stage on the onset of this disease. Based on our comprehensive integrative bioinformatics analysis, several pathways intermediate a role in T2D development, including insulin signaling pathway. Along with all those candidates, miR135 and the relative targets, especially *Insr*, are among the original top-ranking T2D risk factors. Our data showed that *Insr* gene expression was significantly down-regulated in C2C12 cells after transfection with miR-135. Increased expression of the miR-135 had accompanied with decline in the expression of *Insr*, as the first component of the insulin signaling pathway, compared to the control cell line. However, *Vamp2* was not significantly changed after transfection with miR-135. Since we had observed an inverse expression pattern of miR-135 and *Insr* in the transfected C2C12 cells, our team sought to identify whether it has any effect on glucose uptake. Interestingly, the miR-135 transfected cells showed a down-regulation of *Insr* gene expression accompanied with a significant decrease in glucose uptake when compared to PCC, while it was the same as IRC. We determined that reduction of *Insr* expression level in the presence of miR-135, contributed to the downstream effects of insulin action. *INSR* is a critical component of the insulin signaling cascade .Therefore, abnormality of *INSR* expression appears to be a significant defect that may result in reduction of insulin-stimulated glucose disposal in muscle. To confirm our results, we compared them with Affymetrix cDNA microarray data obtained from GEO accession numbers GSE6798 and GSE26168. Skov et al. (31) had previously analyzed 13 different RNA samples from healthy control and 16 different RNA samples from obese human insulin resistance vastus lateralis muscle (overall 29 microarrays, GEO accession # GSE6798). These individuals were selected from a larger cohort who participated in a previously reported study. In the other study, Karolina et al. (32), carried out mRNA microarray investigation on 8 different peripheral blood RNA samples of T2D individuals and 9 control samples (GEO accession # GSE26168). Microarray analysis confirmed our findings (P≤0.05) except for *VAMP2*. However, the magnitude of microarray data changes was not similar to qRTPCR results. Regarding that qRT-PCR indicator is linear over a wide concentration series (35), as completed by serial dilution tests with different samples (data not shown), we believe that this technique provides a more accurate representation of changes in the level of specific transcripts in the miR-135 transfected versus the normal C2C12 cells, compared to the microarray analysis. Never the less, both studies indicated that there is a significant down-regulation of *INSR* expression pattern in insulin resistance and T2D samples. 

According to our main results, miR-135 overexpression has negative effects on glucose uptake seemingly through targeting *Insr* in the insulin signaling pathway. It gives the impressions that overexpression of miR-135 could be involved in insulin resistance, and it could affect related pathways responding normally to insulin using signaling molecules involved in removing glucose in tissues, through down-regulation of *Insr*. Consistent with our finding, Agarwal et al. (36) found that miR-135 level was elevated in diabetic skeletal muscle and miR-135 silence *in vivo* improved glucose tolerance via downregulation of IRS2, the other member of insulin signaling cascade. Targeting different components of one signaling pathway by a single miR implicates the regulatory role of respective miR (37,38). Hence, our results accompanied with Agawal’s finding further confirming the involvement of miR-135 in insulin resistance. 

## Conclusion

In this study, it was demonstrated that the lack of *Insr* expression and increased level of miR-135, contributed to the poor stimulation of glucose uptake by insulin in C2C12 cell line developing insulin resistance phenotype. However, further studies are required to address complex regulatory roles of this miR. Establishing the role and regulation of muscle miRs will enhance our understanding about insulin resistance development. Combining informatics, biochemical and genetic approaches not only will lucid the miR regulatory role in T2D, but also will raise new opportunities for therapeutic intervention in this complex diseases by identifying candidate miRs as potential targets for clinical application. 
